# Highly Stretchable, Strain-Sensitive, and Ionic-Conductive Cellulose-Based Hydrogels for Wearable Sensors

**DOI:** 10.3390/polym11122067

**Published:** 2019-12-11

**Authors:** Ruiping Tong, Guangxue Chen, Junfei Tian, Minghui He

**Affiliations:** 1 State Key Laboratory of Pulp and Paper Engineering, South China University of Technology, Guangzhou 510640, China; tongpinp@163.com (R.T.); fejftian@scut.edu.cn (J.T.); 2 Guangdong Engineering Research Center for Green Fine Chemicals, Guangzhou 510640, China

**Keywords:** stretchability, strain sensitivity, cellulose-based hydrogels, ionic conductivity, wearable sensors

## Abstract

To extend the applications of natural polymer-based hydrogels to wearable sensors, it is both important and a great challenge to improve their mechanical and electrical performance. In this work, highly stretchable, strain-sensitive, and ionic-conductive cellulose-based hydrogels (CHs) were prepared by random copolymerization of allyl cellulose and acrylic acid. The acquired hydrogels exhibit high stretchability (~142% of tensile strain) and good transparency (~86% at 550 nm). In addition, the hydrogels not only demonstrate better sensitivity in a wide linear range (0–100%) but also exhibit excellent repeatable and stable signals even after 1000 cycles. Notably, hydrogel-based wearable sensors were successfully constructed to detect human movements. Their reliability, sensitivity, and wide-range properties endow the CHs with great potential for application in various wearable sensors.

## 1. Introduction

Smart and sensitive wearable devices that can transduce mechanical deformation into electrical signals [1,2] have attracted great interest in the fields of personalized health monitoring [3,4], electronic skins [5], and human motion detection [6], among others. The development of sensitive wearable devices has created high demand for flexible soft materials with high stretchability, strain sensitivity, and ion transport [2,7]. Notably, hydrogels, a novel kind of soft material consisting of cross-linked networks of hydrophilic polymers in water, have attracted worldwide attention for their liquid-like transport and solid-like mechanical properties [8]. Especially, ionic conductive hydrogels that are swollen with electrolyte solutions [7,9,10] have high transparency and have drawn particular interest [11,12]. There are two categories of hydrogels including synthetic polymer- and biopolymer-based hydrogels [13]. Noticeably, the biopolymer-based hydrogels have acquired increasing attention due to their biodegradability, safe nature, and inexhaustibility, among other properties [13,14]. Of the biopolymers, cellulose is the most abundant and has great potential application in the preparation of hydrogels [13,15,16].

However, cellulose-based hydrogels, especially pure-cellulose-based hydrogels, are usually lacking in mechanical properties (e.g., mechanical strength and stretchability) [15,17,18], which would result in unstable electrical behavior [7,15,19,20,21]. To improve the mechanical performance of cellulose-based hydrogels, many studies have been done. For example, Zhao et al. constructed high-strength and high-toughness cellulose hydrogels by chemical cross-linking with epichlorohydrin followed by physical cross-linking induced by an ethanol solution [15]. In our previous study, ultra-stretchable cellulose ionic hydrogels were prepared by using (NH_4_)_2_S_2_O_8_ to initiate free radical polymerization, then using NaCl to induce physical cross-linking [22]. However, although high mechanical performance was obtained, there remains the challenge of combining the properties of transparency, high sensitivity, and high mechanical performance to construct wearable sensors that imitate the human skin (with 2–80 KPa of modulus for the dermis and 140–600 KPa of modulus for the epidermis) [23,24,25]. Cellulose-based hydrogels in which cellulose is the main scaffold and a synthetic polymer is blended in the system have become a notably attractive option in recent years, because they have many advantages, such as adjustable mechanical properties [19,26,27]. This provided us with a new idea to explore and improve the mechanical properties of cellulose-based hydrogels for wearable sensors.

**Figure 1:**
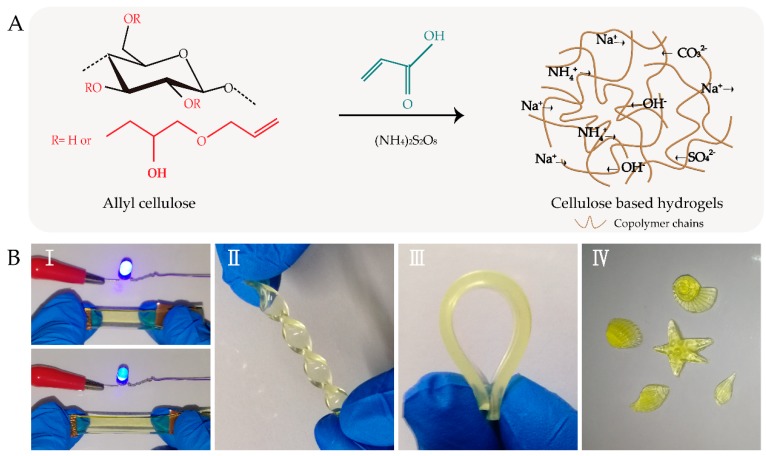
(**A**) Schematic illustration of the preparation of cellulose-based hydrogels (CHs). (**B**) The achieved hydrogels were (I) ionic-conductive and stretchable, (II) twistable, (III) flexional, and (IV) could be molded into various forms including seashell, starfish, and conch shapes.

Herein, highly stretchable, strain-sensitive, and ionic-conductive cellulose-based hydrogels (CHs) have been prepared by random copolymerization of allyl cellulose and acrylic acid, as shown in Figure 1. The acquired hydrogels exhibit high stretchability (with strain up to ~142%) and good transparency (~86% at 550 nm). In addition, the hydrogels demonstrate better sensitivity in the wide linear range (0–100%). The hydrogels also exhibit excellent repeatable and stable signals, even after 1000 cycles. Notably, hydrogel-based wearable sensors have been successfully constructed to detect human movements.

## 2. Materials and Methods

### 2.1. Chemicals and Materials

Cellulose (C104842), allyl glycidyl ether (A106314), and ammonium persulfate (A112447) were provided by Aladdin Reagent Co., Ltd. (Shanghai, China). Acrylic acid was a chemically pure reagent and was provided by Tianjin Kemiou Chemical Reagent Co., Ltd. (Tianjin, China). Sodium hydroxide, ether, acetone, and urea were analytical reagents and were provided by Guangzhou Chemical Reagent Factory (Guangdong, China).

### 2.2. Synthesis of Allyl Cellulose

The synthesis of allyl cellulose was performed following a previously published method [22]. Briefly, to obtain a transparent 6 wt% cellulose solution, the cellulose was dissolved in 7 wt% NaOH/12 wt% urea solution at a temperature under −12.5 °C. Then, allyl glycidyl ether (of molar ratio 8.0 to anhydroglucose unit of cellulose) was added dropwise into the transparent cellulose solution, under a nitrogen atmosphere, at 30 °C for 24 h. Thereafter, the residual allyl glycidyl ether in the mixture was removed by thorough washing with ether, then the mixture was further rotary-evaporated for 1 h at 30 °C. Additionally, for the structural characteristics of allyl cellulose, the mixture was thoroughly washed with acetone, then the purified sample was obtained by further dialyzing for 3 days [22].

### 2.3. Fabrication of Cellulose-Based Hydrogels

To prepare cellulose-based hydrogels, acrylic acid (0–5.0 wt%) and 1.0 wt% of ammonium persulfate were added to the allyl cellulose solution while extensively stirring. Thereafter, the mixture was poured into a laboratory-made mold and maintained under 30 °C for 24 h. The obtained cellulose-based hydrogel was denoted as CHx, representing x wt% acrylic acid.

### 2.4. Characterization

Attenuated total reflection Fourier transform infrared (ATR–FTIR spectra were recorded using a VERTEX 70 ATR–FTIR spectrometer (Bruker, Karlsruhe, Germany). XRD patterns were recorded using a Bruker D8 diffractometer. The transmittance spectrum was obtained using a UV−vis spectrometer (Cary60, Agilent Technologies Inc., Palo Alto, CA, USA). Scanning electron microscopy (SEM, EVO 18, Carl Zeiss AG, Jena, Germany) was carried out to observe the surface morphology of CHs. Mechanical performance tests were conducted using a tensile–compressive machine (Instron 5565, Instron Corporation, Canton, MA, USA), with a speed of 10 mm/min for the tensile test and 5 mm/min for the compressive test. The conductivity of CHs was measured on a CHI660E electrochemical workstation (Chenhua Instrument Co. Ltd., Shanghai, China). The resistance variations were recorded using a DMM7510 digital graphical sampling multimeter (Keithley Instruments, Cleveland, OH, USA).

The CHs were immersed in deionized water for 3 days at room temperature and dried in a vacuum at a temperature under 50 °C to evaluate their swollen ratios, which were calculated as follows [7]:

(1) $$\mathrm{Swollen}\;\mathrm{ratio}=\frac{W_{\mathrm{wet}}-W_{\mathrm{dry}}}{W_{\mathrm{dry}}}\times 100\%$$

where W_dry_ and W_wet_ represent the weights of the dried and swelling equilibrium hydrogels, respectively.

## 3. Results and Discussion

### 3.1. Fabrication and Structure of Cellulose-Based Hydrogels

Figure 1A shows the fabrication of flexible, highly strain-sensitive and ionic-conductive CHs. A moderate dosage of acrylic acid was added into the prepared allyl cellulose solution for improving the mechanical performance of the hydrogels, which would in turn improve the stability of their electrical behavior [20]. Subsequently, the mixture was free-radical-polymerized using ammonium persulfate to initiate the preparation of CHs. Notably, the large dosage of ammonium persulfate would stiffen the hydrogels and damage their mechanical performance [7]. Various kinds of ions appeared in CHs, owing to the presence of NaOH, urea, and ammonium persulfate in the system [28,29]. The obtained hydrogels had ionic conductivity and could work as wires, property which was clearly displayed when the LED indicator gradually dimmed with increasing tensile strain (Figure 1BI). In addition, the CHs were flexible and could withstand various deformations (Figure 1BⅡ, Figure 1BⅢ). Furthermore, the CHs could be molded into a series of forms such as seashell, starfish, and conch (Figure 1BⅣ).

**Figure 2:**
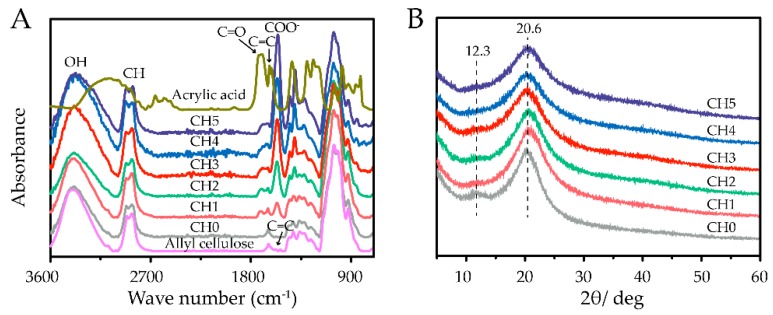
(**A**) ATR–FTIR spectrum of acrylic acid and cellulose-based hydrogels with different weight ratios of acrylic acid. (**B**) XRD profiles of CHs.

To identify and contrast the interactions between allyl cellulose, CHs, and acrylic acid, ATR–FTIR spectroscopy was performed (Figure 2A). The peaks at 1633 and 1570 cm^−1^ were attributed to C=C stretching [30,31]. The characteristic peaks at 1718 cm^−1^ correspond to the C=O bond of COOH [31,32]. Additionally, the peaks at 1560 cm^−1^ are related to the stretching of COO^-^ groups ascribed to the deprotonation of carboxylic acid groups under alkaline conditions [31]. Allyl cellulose was successfully synthesized as indicated by the appearance of C=C stretching at 1570 cm^−1^. Obviously, with the addition of acrylic acid, the characteristic peak at 1560 cm^−1^ (related to COO^-^ groups) appeared in the hydrogels, and the peak’s intensity increased with the increasing dosage of acrylic acid. Moreover, the disappearance of C=C stretching in CHs sufficiently demonstrated that copolymerization of allyl cellulose and acrylic acid occurred. To understand the structural changes during the preparation of the CHs, XRD analysis was introduced (Figure 2B). The XRD patterns of CHs exhibited two peaks located at 12.3° (110) and 20.6° (200), which were the reflection of cellulose II crystallite [33]. This result indicated that the addition of acrylic acid had nonsignificant influence on the crystal form of the hydrogels.

### 3.2. Transparency and Swelling Properties

**Figure 3:**
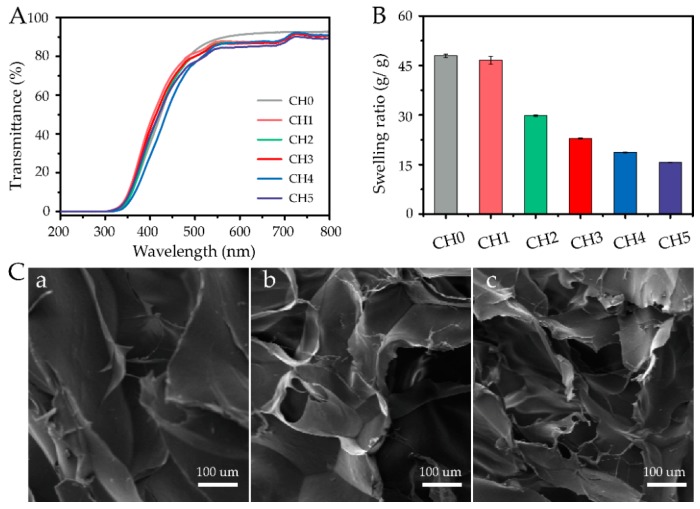
(**A**) UV spectrum of cellulose-based hydrogels (2 mm thickness) with different weight ratios of acrylic acid. (**B**) Swelling ratios of CHs. (**C**) SEM images of the swollen hydrogels (a) CH1, (b) CH3, and (c) CH5.

The highly transparent sensory sheets did not impede an optical signal, which is essential for invisible and optoelectronic devices [34]. As shown in Figure 3A, the transparency of CHs was studied. At a typical wavelength (550 nm), there was a trend of reduced transparency with increased acrylic acid dosage, and the values of transparency were 88.93%, 87.46%, 86.69%, 86.23%, 85.76%, and 83.94%, respectively. This result was most likely due to the fact that the higher the dosage of acrylic acid, the denser the network structures. The denser network structures would prevent the passing of light [7].

The water adsorption properties of hydrogels play an important role in various technological and biomedical applications [35]. Water adsorption mainly depends on network density [36,37]. Thus, the swelling ratios were evaluated to study the structural characteristics of hydrogels (Figure 3B). The swelling ratios of CHs displayed a reduced trend as the dosage of acrylic acid increased. The values of the swelling ratios were 47.95 g/g, 46.62 g/g, 29.81 g/g, 22.93 g/g, 18.68 g/g, and 15.64 g/g, for increasing doses of acrylic acid. This result might be ascribed to the copolymerization of allyl cellulose with acrylic acid resulting in denser networks. To further explore the structural characteristics of hydrogels, the microstructures of swollen hydrogel cross sections were examined using SEM, as shown in Figure 3C. Obviously, the microstructures became denser with the increase of acrylic acid dosage, which implied a gradual decrease in water absorption and swelling capacity. These results further indicated that the larger the dosage of acrylic acid added, the denser the hydrogels’ structure became.

### 3.3. Mechanical Performance

**Figure 4:**
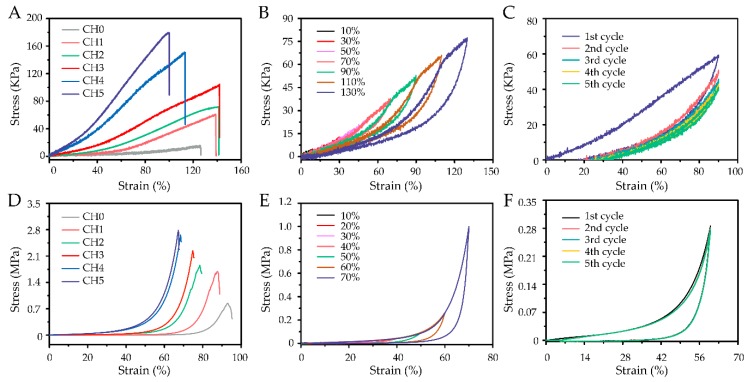
Mechanical properties of cellulose-based hydrogels with different weight ratios of acrylic acid. (**A**) Tensile and (**D**) compressive stress−strain curves of CHs. Tensile stress–strain curves of CH3 (**B**) with varying maximum stretching and (**C**) for the first to the fifth cycles at 90% strain under loading–unloading cycles. Compressive stress−strain curves of CH3 (**E**) with varying maximum compression and (**F**) for the first to the fifth cycles at 60% strain under loading–unloading cycles.

The cross-linked density strongly affected the hydrogels’ mechanical properties [7]. A series of tensile and compressive tests were performed to quantitatively evaluate the mechanical performance of the hydrogels, as shown in Figure 4 and Table 1.

Under tension, the tensile strength and Young’s modulus (tensile modulus) of CHs increased from 15.50 KPa and 12 KPa to 178.43 KPa and 103 KPa, respectively (Figure 4A, Figure S1A). The tensile strain first increased and then decreased, and the CH3 had the highest tensile strain (141.86%). The high dosage of acrylic acid significantly contributed to a high network density, resulting in stiffness and damaging the hydrogels’ tensile performance. Thus, a moderate network density induced by the copolymerization of allyl cellulose and acrylic acid contributed to the acquisition of remarkable mechanical performance by the CHs. In addition, low-modulus sensors would more easily detect micro strains of the skin, and the combination of high stretchability and low modulus would make hydrogels well adaptable to the epidermis [23]. Obviously, CH3 had a low tensile modulus (35 KPa) compared to human skin (2–80 KPa of modulus for the dermis, 140–600 KPa of modulus for the epidermis). Cyclic loading–unloading tests with varying maximum stretching were applied, and the CH3 with high stretchability was taken as representative, as shown in Figure 4B. The tensile stress–strain curves of CH3 with varying maximum stretching showed that the hysteresis loop steadily increased, which was due to energy dissipation through the dissociated hydrogen bonding, endowing the hydrogels with excellent mechanical performance [38]. Moreover, negligible strength degradation and plastic deformation of hydrogels even at a high strain of 130% were observed. This result implied that the hydrogels could be adapted to various amounts of continuous strains. Additional loading–unloading tests were then carried out to evaluate the antifatigue properties of CH3. As shown in Figure 4C, a distinct hysteresis loop was observed in the first cycle, and a large drop in stress appeared in the second cycle. This phenomenon indicated that most of the material softening and hysteresis occurred during the first cycle. Moreover, the stress–strain curves after the second cycle were almost overlapping, which implied that the CH3 reached a steady state.

Under compression, the effect of thickness on the compressive behavior of the hydrogels was also studied, as shown in Figure S2. CH0 with small thickness (2 mm) displayed higher mechanical performance, which was more likely attributed to the fact that any minor crack would eventually degrade a sample’s performance. Therefore, 2 mm was the thickness that was further studied unless otherwise specified. As the dosage of acrylic acid increased, the modulus and compressive strength drastically increased from 3 KPa and 827 KPa to 236 KPa and 2789 KPa, respectively (Figure 4D, Figure S1B). However, the fracture strain decreased from 92.66% to 67.17%. Fortunately, CH3 also had a low modulus (59 KPa) of compression. Significantly, the loading and unloading curves with various maximum compression values overlapped those of the previous cycles (Figure 4E), which indicated that the hydrogel had an excellent shape memory. As shown in Figure 4F, CH3 also displayed high compression stability such that the compression strain curves of the hydrogel almost overlapped after five cycles at 60% strain.

### 3.4. Electrical Performance and Integration with Wearable Sensors

**Figure 5:**
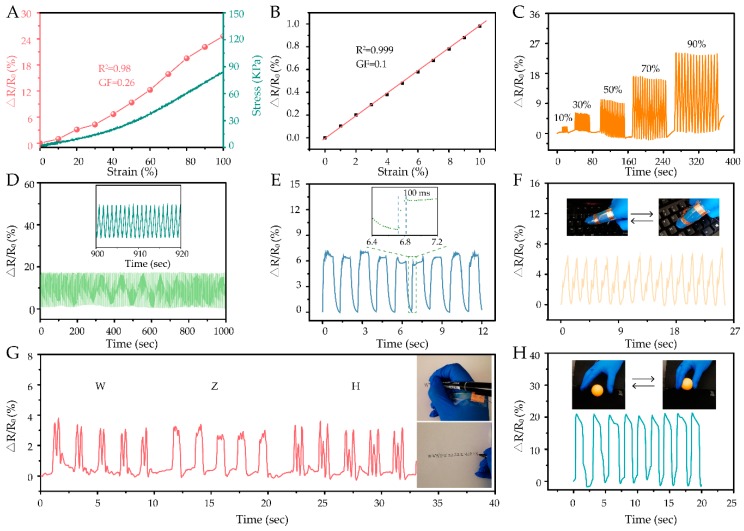
Sensory and electrical performances of the cellulose-based hydrogel CH3. (**A**) Resistance change ratio variations (ΔR/R_0_) and stress versus tensile strain. (**B**) Linear relationship between ΔR/R_0_ and strain, with a gauge factor (GF) within 10% of tensile strain. (**C**) ΔR/R_0_ at different tensile strains. (**D**) Fatigue resistance at 70% tensile strain for 1000 cycles, at a speed of 10 mm/s. (**E**) ΔR/R_0_ response to manual loading–unloading of 995 Pa. The response of the strain sensor for monitoring biosignals corresponding to (**F**) tapping the keyboard, (**G**) writing, and (**H**) grasping.

The CHs displayed ionic conductivity because of the presence of NaOH, urea, and ammonium persulfate in the system, which made it possible to build wearable sensors. As shown in Figure S3, the conductivities of CHs decreased from 0.16 mS cm^−1^ (CH0) to 0.036 mS cm^−1^ (CH5). The higher dosage of acrylic acid caused higher network density, which would prevent the ions from moving [7]. Considering that mechanical, optical, and conductive properties are important for wearable sensors, CH3 with the highest tensile strain (141.86%) and moderate optical (86.23%) and conductive properties (0.05 mS cm^−1^) was used for further evaluation, as shown in Figure 5.

As shown in Figure 5A, the resistance change ratio variation (ΔR/R_0_) of CH3 displayed a highly linear relationship (R_0-100%_^2^ = 0.980), and the gauge factor (GF, representative of sensitivity) [3,39] was 0.26, which implied that CH3 could serve as a wide-range and highly reliable tensile sensor. More importantly, this sensor could also reliably detect a tiny strain of 10%, because its highly linear relationship between resistance change ratio variation and strain (R_0-10%_^2^ = 0.999) and good GF (0.1), as shown in Figure 5B. Figure 5C shows the ΔR/R_0_ responses of CH3 to different tensile strains. The ΔR/R_0_ quickly rose with tension and decreased accompanying release, which indicated that the hydrogel was highly sensitive. In addition, the intensity of ΔR/R_0_ increased with the increase of tension strain, which suggested that the hydrogel could effectively distinguish different strain levels. As shown in Figure 5D, the strain sensor also displayed excellent stability at 70% strain for 1000 cycles. To prevent water evaporation from the hydrogel, elastomeric and very high bond tapes (VHB 4905, Minnesota Mining and Manufacturing, Saint Paul, MN, USA) were applied in the stability test [7,40].

Hydrogels with high stretchability and excellent, reliable sensitivity could be employed as touch or wearable sensors. The hydrogel here examined displayed fast response time (100 ms), when a load (995 Pa) was quickly applied on it (Figure 5E). In addition, the hydrogel was wrapped with transparent tape and attached to the experimenter’s hand in different positions to detect hand’s movements while tapping a keyboard (Figure 5F), writing (Figure 5G), and grasping (Figure 5H). The response signals were repeatable and stable for the same motion. Moreover, the shapes and signals’ intensity were distinguishable for each respective motion. Thus, the hydrogel could be used in wearable devices, for its high reliability and sensitivity.

## 4. Conclusions

Herein, we reported highly stretchable, strain-sensitive, and ionic-conductive CHs prepared by the random copolymerization of allyl cellulose and acrylic acid in NaOH/urea aqueous solution. The acquired hydrogels exhibited high stretchability (with strain up to ~142%) and good transparency (~86% at 550 nm). Moreover, the hydrogels not only demonstrated a better sensitivity in a wide linear range (0–100%) but also exhibited excellent repeatable and stable signals even after 1000 cycles. Significantly, hydrogel-based wearable sensors were successfully constructed to detect human movements. This work offers an exciting method to fabricate functional hydrogels with promising potential for detecting biosignals.

## Figures and Tables

**Table 1:** Conditions and physical properties of CHs.

Samples	Acrylic Acid (wt%)	Compression			Tension			Swelling Ratios (g g^−1^)
		σ_comp_^(a)^ (Kpa)	ε_comp_^(b)^ (%)	E_comp_^(c)^ (Kpa)	σ_Tens_^(d)^ (Kpa)	ε_Tens_^(e)^ (%)	E_Tens_^(f)^ (Kpa)	
CH0	0	827	92.66	3	15.50	126.02	12	47.95
CH1	1	1680	87.67	9	59.86	138.89	14	46.62
CH2	2	1849	78.49	40	71.8	141.50	16	29.81
CH3	3	2216	74.99	59	104.67	141.86	35	22.93
CH4	4	2655	68.50	197	151.22	112.74	76	18.68
CH5	5	2789	67.17	236	178.43	99.82	103	15.64

^(a)^, ^(b)^, and ^(c)^ stress at fracture, fracture strain, and modulus under compression; ^(d)^, ^(e)^, and ^(f)^ tensile strength, elongation at break, and Young’s modulus under tension.

## Supplementary Materials

The following are available online at https://www.mdpi.com/2073-4360/11/12/2067/s1, Figure S1: (A) Young’s modulus and (B) modulus of cellulose-based hydrogels (CHs) with different weight ratios of acrylic acid under tension and compression mode, respectively. Figure S2: Compressive stress−strain curves of CH0 with different thicknesses. Figure S3: Conductivity values of CHs.
