# Supplementary material for: Highly Stretchable, Strain-Sensitive, and Ionic-Conductive Cellulose-Based Hydrogels for Wearable Sensors
Source: Polymers (Basel). 2019 Dec 11;11(12):2067. doi: 10.3390/polym11122067 (PMC6960870; doi:10.3390/polym11122067)
Supplement: Supplementary file 1 [file polymers-11-02067-s001.pdf]

Highly stretchable, strain sensitive and ionic conductive cellulose based hydrogel for wearable sensors

Ruiping Tong [1], Guangxue Chen [1,*], Junfei Tian [1] and Minghui He [1,2,*]

[1] State Key Laboratory of Pulp and Paper Engineering, South China University of Technology, Guangzhou 510640, China; tongpinp@163.com (R.T.); chengx@scut.edu.cn (G.C.); fejftian@scut.edu.cn (J.T.); heminghui@scut.edu.cn (M.H.)

[2] Guangdong Engineering Research Center for Green Fine Chemicals, Guangzhou 510640, China

* Correspondence: chengx@scut.edu.cn (G.C.); heminghui@scut.edu.cn (M.H.)

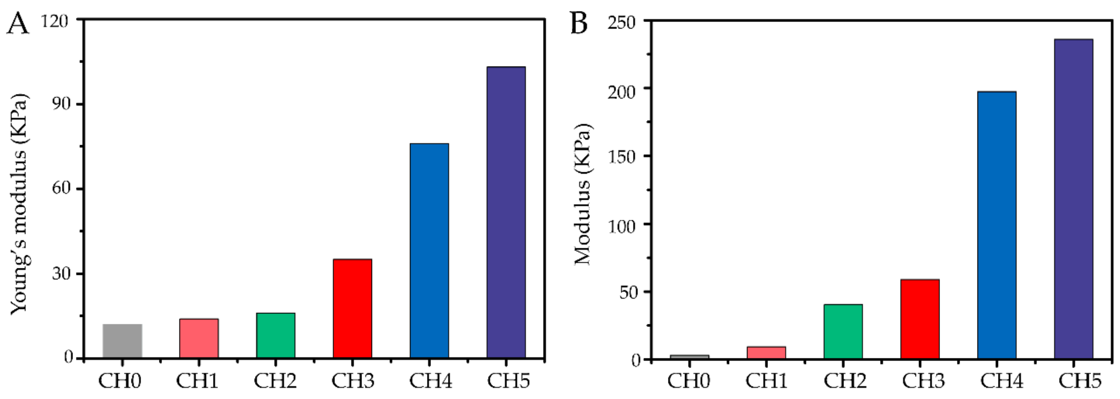

Figure S1 A) Young's modulus and B) modulus of cellulose based hydrogels (CHs) with different weight ratios of acrylic acid under tension and compression mode, respectively.

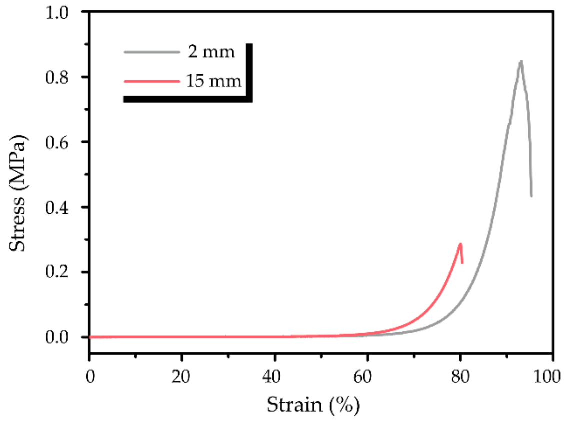

Figure S2 Compressive stress−strain curves of CH0 with different thickness.

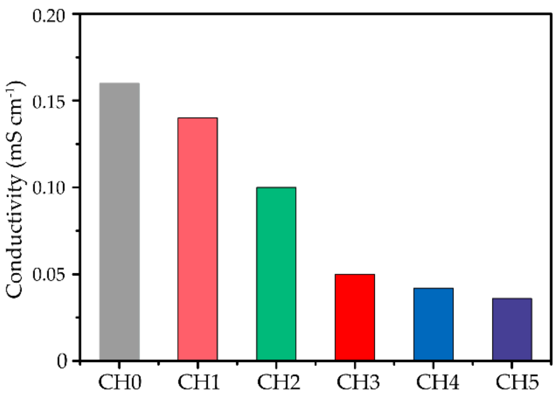

Figure S3 Conductivity values of CHs.
